# Whole-genome sequencing of four culturable endophytic bacteria from German hardneck garlic cloves (*Allium sativum* L.)

**DOI:** 10.1128/mra.01225-23

**Published:** 2024-03-12

**Authors:** Girish Kumar, Han Ming Gan, Peter Wengert, Trevor Penix, Anutthaman Parthasarathy, André O. Hudson, Michael A. Savka

**Affiliations:** 1 Thomas H. Gosnell School of Life Sciences, Rochester Institute of Technology, Rochester, New York, USA; 2 Department of Biological Sciences, Sunway University, Petaling Jaya, Malaysia; 3 Patriot Biotech Sdn. Bhd., Subang Jaya, Malaysia; 4 School of Chemistry and Biosciences, University of Bradford, West Yorkshire, United Kingdom; University of California Riverside, Riverside, California, USA

**Keywords:** *Allium sativum*, German hardneck garlic cloves, endophyte genomes, disease resistant, pathogenicity

## Abstract

We present the whole-genome sequence of four bacterial endophytes associated with German hardneck garlic cloves (*Allium sativum* L.). Among them, *Agrobacterium fabrum* and *Pantoea agglomerans* are associated with plant protection, while *Rahnella perminowiae* and *Stenotrophomonas lactitubi* are pathogens. These data will facilitate the identification of genes to improve garlic.

## ANNOUNCEMENT

Endophytes play a vital role in plant growth, development, and stress defense without causing noticeable changes or infection (1). Garlic (*Allium sativum* L.) is a significant crop renowned for its culinary and medicinal properties and as a source of endophytic microorganisms (2). In recent studies, bacterial endophytes that are linked with garlic roots (3) and black garlic processing (4) have been isolated and identified. However, the isolation, identification, and whole-genome sequencing of bacterial endophytes from hardneck garlic cloves have not been attempted previously. In this research, we have successfully isolated and sequenced four bacterial endophytes that are associated with garlic cloves.

Mature field grown hardneck German garlic bulbs were obtained from Bischoping Farm, Rush (Longitude: −77.6374368; Latitude: 43.01495449999999), New York in August. The cloves were separated from the bulbs, peeled and subjected to surface sterilization using a 20% sodium hypochlorite (NaOCl) solution, followed by two consecutive rinses with sterile distilled water for the isolation of cultivable bacterial endophytes. The sterilized cloves were axenically chopped and then introduced into tryptic soy broth (TS) liquid medium and incubated at 28°C for 3 days. The resulting bacterial culture was 10-fold serial diluted and then plated onto TS agar media and incubated under identical conditions. Subsequently, four separate single colonies with distinct morphologies were obtained and subcultured on TS agar medium and incubated at 28°C for 48 hours to ensure strain purity.

The E.Z.N.A. bacterial DNA kit (Omega Bio-Tek, Norcross, GA, USA) was used to extract genomic DNA from 25 mg of pelleted cells. The DNA concentrations were then measured with a Qubit 3.0 fluorometer (Life Technologies, MD, USA). Subsequently, sequencing libraries were created following the Nextera XT library preparation protocols using 0.2 ng/µL of DNA and sequenced on the MiSeq sequencer (Illumina, San Diego, CA, USA) utilizing a MiSeq reagent V3 kit (2 × 300 cycles), following the manufacturer’s guidelines at the Genomics Lab, Rochester Institute of Technology. Raw reads were subjected to quality control processing with the fastp version 0.23.2 (5) and then *de novo* assembled into contigs using the SPAdes genome assembler (6) (version 3.15). The NCBI Prokaryotic Genome Annotation Pipeline (7) (version 6.5, http://www.ncbi.nlm.nih.gov/genome/annotation_prok/) was used to automatically annotate the genomes upon upload. Default parameters were used for all software unless otherwise specified.

**Fig 1:**
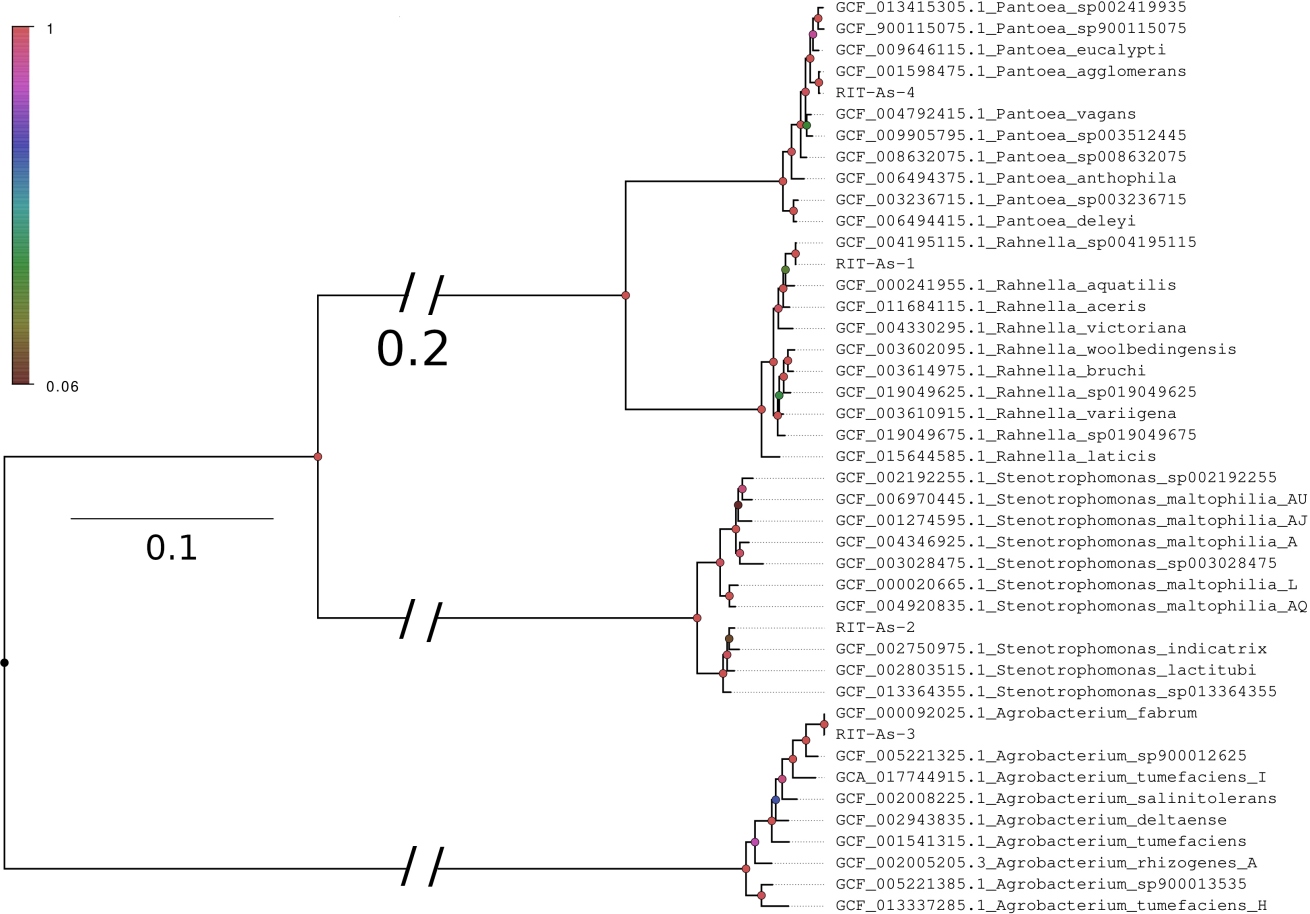
Maximum likelihood tree depicting the evolutionary relationships among the isolated garlic endophytes (blue tip label) and their closely related representative strains. The tree was constructed using FastTree version 2.1.10 (12) based on the concatenated alignments of highly conserved Proteobacteria proteins produced from the GToTree v1.7.07 pipeline (13). Briefly, the pipeline identified and aligned a total of 119 single-copy proteins from each genome annotation using hmmsearch v 3.3.2 (14) and muscle v 3.8.1551 (15), respectively. Nodes were colored according to their SH-like support values, and branch lengths indicate the number of substitutions per site. Branches leading to the major taxa are shortened to improve readability.

All four endophyte species were phylogenetically placed into their respective genera clades in the constructed maximum likelihood tree, corroborating with their initial taxonomic assignment based on average nucleotide identity (ANI; Fig. 1). Of the four genomes sequenced (Table 1), two bacterial endophyte species (*Agrobacterium fabrum* and *Pantoea agglomerans*) confer protection to plants against abiotic stress (8, 9). On the other hand, endophytes *Rahnella perminowiae* and *Stenotrophomonas lactitubi* have been found to be pathogens (10, 11). The whole-genome sequencing data presented in this study can assist in identifying genes associated with endophyte-mediated resistance and pathogenicity in garlic, thereby contributing to the progress of garlic cultivation.

**TABLE 1:** Information on genome annotation of four bacterial endophytes associated with garlic (*Allium sativum* L.) cloves^*a*^

Sample	No. of raw reads	SRA accession	Assembly accession	Assigned taxonomy	Reference genome with accession number	% ANI	% cov	Assembly size (bp)	Coverage (×)	No. of contigs	*N*_50_	%GC	No. of genes
RIT-As-1	53,314,682	SRR22245962	GCA_026241995.1	*Rahnella perminowiae*	GCA_019049755.1	99.28	91.23	5,449,955	10.62	534	16,306	51.56	5,376
RIT-As-2	53,672,656	SRR22245961	GCA_026242005.1	*Stenotrophomonas lactitubi*	GCA_002803515.1	95.54	85.69	4,527,262	12.22	245	28,938	66.06	4,267
RIT-As-3	269,038,734	SRR22245960	GCA_026241955.1	*Agrobacterium fabrum*	GCA_000092025.1	99.99	99.96	5,250,432	47.41	33	314,096	59.15	4,967
RIT-As-4	246,616,136	SRR22245959	GCA_026241965.1	*Pantoea agglomerans*	GCA_003710245.1	98.74	89.72	4,974,910	71.38	28	451,856	54.84	4,669

^*a*^ %ANI, percent average nucleotide identity; % cov, percent genome query coverage.

## Data Availability

The whole genome sequences reported in this article were deposited in GenBank, and accession numbers for each SRA and whole-genome assembly are available in Table 1.
